# The Pattern of Tooth Loss for Periodontally Favorable Teeth: A Retrospective Study

**DOI:** 10.3390/biology11111664

**Published:** 2022-11-15

**Authors:** Peter Yanni, Donald A. Curtis, Richard T. Kao, Guo-Hao Lin

**Affiliations:** 1 Private Practice, Roseville, CA 95661, USA; 2 Department of Preventive & Restorative Dental Sciences, University of California, San Francisco, CA 94143, USA; 3 Department of Orofacial Sciences, School of Dentistry, University of California, San Francisco, CA 94143, USA

**Keywords:** diagnosis, prognosis, periodontitis, retrospective study

## Abstract

**Simple Summary:**

With the high prevalence of periodontal disease, it is critical to be able to define dental prognosis in order to define treatment strategies for maintaining oral health. While a favorable tooth prognosis was given, some teeth are still extracted soon after being considered favorable. Our study investigated the reasons why these teeth are lost even with a periodontally favorable tooth prognosis. The results demonstrate that previously root-canal-treated teeth present a higher odds ratio for early loss due to caries, recurrent endodontic lesions, or fracture. In addition, patients with anti-depressant medication use, sporadic maintenance, initial probing depths ≥ 5 mm, and furcation involvement represent a significantly higher odds ratio of tooth loss due to periodontal disease even for initially favorable teeth.

**Abstract:**

To retrospectively analyze local and systemic factors that resulted in the short-term tooth loss of teeth that were previously assigned a favorable prognosis in patients who were seen and treated over an observational five-year period. This retrospective study included the records of patients who had a minimum of two dental exams at least twelve months apart over a 5-year period. This study investigated extracted teeth with an initially favorable periodontal prognosis that were then divided into one of four categories based on the reason for extraction: caries, periodontal disease, endodontic reasons, or fracture. Patient- and tooth-related factors associated with the extracted teeth were recorded: crown-to-root ratio, initial pocket depth, initial periodontal diagnosis, maintenance interval, presence of existing restoration, furcation involvement, and systemic conditions. Data analysis was performed using a linear mixed model. A total of 50 patients with 111 teeth met the inclusion criteria for this study. A higher odds ratio (OR) for tooth loss due to caries, endodontic reasons, and fracture were found in teeth with a history of root canal treatment with an OR of 3.61, 3.86, and 2.52, respectively. For tooth loss due to periodontal disease, higher ORs were found in patients who were on anti-depressants (OR = 4.28) and patients who had an initial diagnosis of Stage III/IV periodontitis (OR = 2.66). In addition, teeth with initial probing depths ≥5 mm (OR = 4.32) and with furcation involvement (OR = 1.93) showed a higher OR for tooth loss due to periodontal disease. Within the limitations of this study, previously root-canal-treated teeth present a higher OR for early loss due to caries, recurrent endodontic lesions, or fracture. In addition, patients with anti-depressant medication use, sporadic maintenance, initial probing depths ≥5 mm, and furcation involvement represent a significantly higher OR of tooth loss due to periodontal disease even for initially favorable teeth.

## 1. Introduction

One of the most prevalent and challenging tasks for dental clinicians today is managing and treating patients with periodontitis. In 2012, Eke et al. conducted a study using data from the National Health and Nutrition Examination Survey (NHANES) that examined the prevalence, severity, and extent of periodontitis in adults in the United States [1]. In this study, they found that in over 45% of the sample, approximately 65 million people, had periodontitis. When examining the severity of the disease, they found that moderate and severe cases of periodontitis represented a combined 38.5% of this sample. Given the prevalence of periodontal disease, it is critical to be able to define dental prognosis in order to define treatment strategies for maintaining oral health. Determining an accurate prognosis of teeth over time has proven to be a challenging task for clinicians and has direct implications on treatment planning and patient acceptance [2]. Several prognostication systems have been developed that attempt to aid practitioners in predicting the long-term outcomes of teeth [2,3,4,5,6,7]. 

One of the earliest periodontal prognostication systems was elucidated by Hirschfeld and Wasserman in 1978 [3]. This prognostication system divided patients into well-maintained, downhill, and extreme downhill based on teeth lost throughout an average follow-up period of 22 years in a private practice setting. In addition, teeth were categorized as favorable, questionable, or hopeless depending on several local factors. These factors included furcation involvement, non-eradicable pockets, extensive alveolar bone loss, and marked mobility. The data showed that 31.3% of questionable teeth were lost during the follow-up period.

Later on, Becker et al. proposed a new prognostication system based on a series of studies conducted in a private practice [4,8,9]. These studies examined three categories of patients: treated with maintenance, treated without maintenance, and untreated periodontal disease. They further proposed a prognostication system on the basis of several clinical and radiographic findings. Teeth were categorized as either questionable or hopeless based on specific findings, such as extent of alveolar bone loss, pocket depths, furcation involvement, and tooth decay, amongst other things. Despite the extensive analysis, the authors expressed uncertainty in determining a prognostication system for hopeless teeth, since several factors, such as patient comfort, restorative treatment plans, and conditions of adjacent teeth, should be considered [9].

To remedy the concerns with past prognostication systems, McGuire proposed a detailed system that examined several factors and included both tooth and overall prognosis [5]. The classification was based on tooth mortality and was comprised of the following categories: good, fair, poor, questionable, and hopeless. While this prognostication system proved to be predictable for teeth designated with a good or hopeless prognosis, it often fell short for teeth deemed poor or questionable [6]. The accuracy was reported to be 81% with a follow-up of 5–8 years, but dropped significantly to 50% when teeth with a good prognosis were excluded [2,6].

In an attempt to further develop a more accurate prognostication system, Kwok and Caton developed a system to predict the long-term outcomes of teeth by taking into account both local and systemic factors that may affect tooth stability [2]. This system was developed with three key tenets in mind: achieving the periodontal stability of supporting tissues rather than tooth mortality, the timing of the projection and continual re-evaluation, and the consideration of individual teeth versus the overall dentition [2]. Using these principles, Kwok and Caton proposed four categories of prognosis: favorable, questionable, unfavorable, and hopeless. A favorable prognosis is defined as having a periodontal status that can be stabilized with comprehensive periodontal treatment and maintenance. Teeth are regarded as questionable when there are local and systemic factors that may or may not be controlled with treatment and patient motivation. However, their stability declines when local and/or systemic influences become uncontrolled. Unfavorable teeth are likely to breakdown and achieving stability is not expected even with comprehensive periodontal treatment. Finally, hopeless teeth are those that must be extracted [2].

A recent study conducted by our team at the University of California San Francisco (UCSF) School of Dentistry investigated the strengths and the weaknesses of this prognostication system in an attempt to validate its use and to help clinicians develop appropriate short- and long-term treatment plans [10]. The retrospective study reviewed the charts of patients from 2013 to 2019 that had received annual periodic exams and had two separate recorded entries spaced at least 12 months apart. The study included a total of 4046 teeth from 174 patients and found that the tooth survival rate at the latest follow-up for those with an initial favorable, questionable, unfavorable, and hopeless prognosis was 97.9%, 90.7%, 62.5%, and 17.7%, respectively. They concluded that the Kwok and Caton prognostication system can predictably determine tooth survivability within a five-year period.

Interestingly, 2.1% of teeth initially considered favorable were extracted within a five-year follow-up. While the favorable category proved to be predictable, investigating why some of these teeth were extracted so soon after being considered favorable warrants investigation. The loss of favorable teeth, especially early into a patient’s oral prosthetic rehabilitation, can have profound and sometimes deleterious effects on their treatment plan. Therefore, the aim of this study was to retrospectively analyze local and systemic factors that resulted in the short-term loss of teeth that were deemed favorable for patients who were seen and treated at the UCSF School of Dentistry over a five-year period.

## 2. Materials and Methods

### 2.1. Study Design 

This study was designed as a follow-up study to analyze data presented in a previous study [10]. After screening the 830 electronic dental records, 50 patients with 111 teeth met the inclusion criteria for this study. The records were retrieved for these 50 patients who had received annual periodic oral exams and had two separate recorded entries spaced at least 12 months apart, from 2013 to 2021. The inclusion criteria were: (1) teeth presenting with an initial favorable prognosis based on the Kwok and Caton [2] system and (2) teeth that were missing due to extraction at the time of the latest visit. Charts were excluded from the study if the initial prognosis was not favorable or still present at the latest follow-up. Charts were reviewed in chronological order starting from January 2013 to December 2021 by one independent examiner (PY). 

In the current study, the teeth with an initially favorable prognosis, defined as having a periodontal status that can be stabilized with comprehensive periodontal treatment and maintenance, were included due to their short-term loss (within five-year follow-up period). These teeth were divided into one of the four categories based on the reason for extraction. The categories were: caries, periodontal disease, endodontic reasons, and fracture. Teeth that did not have adequate radiographs and/or records to determine the reason for extraction or extracted for orthodontic reasons were excluded. If teeth presented with a combination of the aforementioned ailments, the clinical notes were reviewed to determine the major reason for extraction. If no specific reason was cited, the reviewers placed the tooth into a category based on careful examination of radiographs, periodontal chart, and clinical documentation. Patient information was protected according to the privacy regulations of the Federal Health Insurance Portability and Accountability Act of 1996 (HIPAA). The study protocol was approved by the UCSF Institutional Review Board (ID number: 19-28827).

In addition to the reason for tooth loss, the patient’s demographic data, including gender and age, and medical data, including history of smoking, diabetes, osteoporosis, and use of anti-depressants, were also recorded. Clinical data related to the tooth conditions included: location of tooth within the maxillary or mandibular arch, initial crown-to-root ratio, initial periodontal diagnosis, initial pocket depth, number of dental cleanings per year, presence of existing restoration, presence of existing root canal treatment, and presence of furcation involvement for molar teeth. The mean crown-to-root ratio was determined based on the radiographs using a computer software (MiPACS, Medicor Imaging, Charlotte, NC, USA). Descriptive analyses of the study variables, including mean and standard deviation, were determined. 

### 2.2. Data Analysis

The associations between the reason for tooth extraction and the recorded variables were estimated by a linear mixed model. Odds ratios (ORs) and the 95% confidence intervals (CIs) of the recorded parameters for susceptibility of tooth loss were further calculated. For initial periodontal diagnosis, Stage I and Stage II were used as a baseline reference compared to those with a more severe diagnosis (Stage III and Stage IV). For initial pocket depths, teeth with pocket depths less than 5 mm were used as controls for comparison to those teeth with pocket depths greater than or equal to 5 mm. For the number of dental cleanings per year, those that attended two or more cleaning visits per year served as controls for comparison with those that attended less than two visits. 

Adjustment of the inter-variable influence using multiple linear regression analysis was performed. A *p* value of 0.05 was used as the level of significance. All the statistical analyses were calculated using a computer program (SAS Institute Inc. 2011. Base SAS^®^ 9.3 Procedures Guide, Cary, NC, USA). 

## 3. Results

### 3.1. Patient Demographics

Of the included 50 patients, 52% were male and 48% were female. The mean age of the patients was 57.46, ranging from 30 to 81 years old. In addition, 14% of the patients had a smoking history, 16% had diabetes, 6% had osteoporosis, and 8% used anti-depressant medication. With regard to initial periodontal diagnosis, 8% of patients were classified as Stage I, 20% were classified as Stage II, 64% were classified as Stage III, and 8% were classified as Stage IV. The detailed patient demographic information can be found in Table 1.

### 3.2. Teeth Characteristics

The average follow-up period of the included patients was 3.5 years with a range of 0.33 to 5.3 years. Nearly 60% of the included teeth were from the maxilla, and posterior teeth accounted for 80% of the total teeth. In terms of the reason for tooth loss, caries was the most common reason for extraction (44%), followed by periodontal disease (31%), tooth fracture (14%), and endodontic reasons (10%). The average probing depth for the included teeth was 4.43 mm, ranging from 2 to 9 mm. The initial crown-to-root ratios for teeth extracted due to caries, periodontal disease, endodontic reasons, and fracture were 0.47, 0.48, 0.53, and 0.45, respectively. Teeth extracted due to caries, endodontic reasons, or fracture had mean initial pocket depths of 3.94 mm, 3.91 mm, and 3.82 mm, respectively, whereas periodontally involved teeth had an initial pocket depth of 5.57 mm. For periodontally involved teeth, 100% of the patients were diagnosed with Stage III or IV periodontitis compared to 71%, 55%, and 69% for carious, endodontic, or fractured teeth, respectively. The number of dental cleaning visits per year were 1.28, 1.16, 1.55, and 1.3 for carious, periodontal, endodontic, and fractured teeth, respectively. Existing restoration was observed in 54% of teeth lost for periodontal disease compared to 91% of teeth lost for endodontic reasons, and 50% of teeth lost due to fracture. For lost molars, 31% of them suffered from furcation involvement. The features of the extracted teeth can be found in Table 2.

### 3.3. Risk Indicators Associated with Tooth Loss

No statistical significance could be found for the role of cigarette smoking, diabetes, or osteoporosis in influencing tooth loss for any of the proposed categories. The initial crown-to-root ratio and presence of existing restorations showed no statistically significant influence for any of the categories.

After adjusting for age, gender, and potential inter-variable influence using linear mixed regression analysis, a significantly higher OR for tooth loss due to caries was found in teeth with existing root canal treatment (OR = 3.61, 95% CI = 3.39–4.23). Similarly, a higher OR for tooth loss due to endodontic reasons (OR = 3.86, 95% CI = 3.63–4.09) and root fracture (OR = 2.53, 95% CI = 2.24–2.83) was also found in teeth with a history of root canal treatment. For tooth loss due to periodontal disease, significantly higher ORs were found in patients who were on anti-depressants (OR = 4.28, 95% CI = 4.14–4.42) and patients who had an initial diagnosis of Stage III/IV periodontitis (OR = 2.66, 95% CI = 2.43–2.89). In addition, teeth with an initial probing depth ≥5 mm (OR = 4.32, 95% CI = 3.83–5.12) and with furcation involvement (OR = 1.93, 95% CI = 1.42–2.44) also showed a higher OR for tooth loss due to periodontal disease. The outcome of the statistical analysis can be found in Table 3.

**Figure 1:**
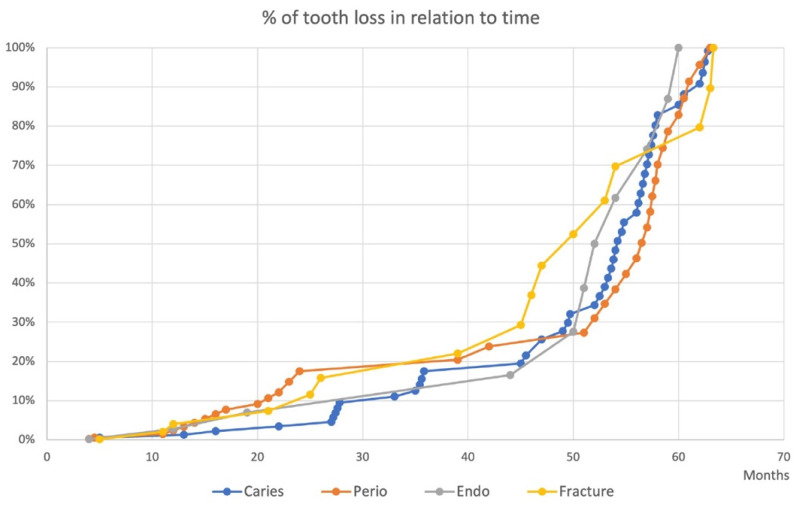
Percentage of tooth loss in relation to time. Each curve illustrates the percentage of tooth loss in relation to time for each individual category. Less than 20% of tooth loss occurred within the first two years and a large number of teeth were lost for all categories after four years of follow-up.

The percentage of tooth loss for each category over time can be seen in Figure 1. Less than 20% of total tooth loss occurred within the first two years after receiving a favorable prognosis. During these first two years, tooth loss due to periodontal disease was slightly higher than the other categories. All categories experienced a spontaneous exacerbation resulting in tooth loss around the 44-month mark.

## 4. Discussion

Assigning a favorable prognosis to a tooth implies a level of confidence that the tooth can remain stable for an extended period of time and provide support to other teeth within the mouth by serving as a retainer for fixed partial dentures, or abutment teeth for removable partial dentures. In addition, favorable teeth can influence irreversible treatment decisions, such as implant placement. Unfortunately, despite a comprehensive analysis of local and systemic factors, favorable teeth are occasionally lost, and this can have potential detrimental effects on treatment planning [10].

While the previous study from our team [10] showed that the Kwok and Caton prognostication system is predictive within a five-year span, 2.1% of the favorable teeth in that study were extracted within five years. The factors influencing the loss of these initially favorable teeth are of clinical significance and warrant further examination so that they can be potentially considered when formulating a tooth prognosis.

Interestingly, in the current study, no statistically significant risk of tooth loss was found for patients with a history of cigarette smoking, diabetes, or osteoporosis, even when looking at teeth lost for periodontal reasons. Since the available evidence supports that uncontrolled diabetes [11,12,13] and cigarette smoking [14,15] are both well-established risk factors for periodontitis that can result in the rapid loss of attachment, one would expect to find that diabetic patients and smokers would be more susceptible to suffering an early loss of favorable teeth due to periodontal disease. The most likely explanation for this finding can be directly attributed to the nature of the Kwok and Caton prognostication system itself, since patients with these risk factors were likely to be given a lower prognosis if the disease status was not likely to be controlled. Patients who were smokers or with a history of diabetes, but had teeth categorized as favorable, were most likely good compliers to the periodontal supportive treatment, resulting in a non-significant association of these risk factors and tooth loss [16].

The analysis of local factors revealed that early tooth loss in the categories of caries, endodontic involvement, and fracture shared a single distinct commonality. For favorable teeth lost due to periodontal disease, a history of root canal treatment did not significantly contribute to tooth loss. Considering that the Kwok and Caton prognostication system was developed specifically for periodontal purposes, it is likely that teeth with root canal treatment that had a certain amount of periodontal involvement were rarely classified as favorable and did not meet the inclusion criteria of this study [2]. Endodontically treated teeth are often restored with posts and/or full coverage restorations that remove substantial internal tooth structure, which may result in a significant correlation with tooth fracture. In an article by Kishen, the author summarized the two main categories of tooth fracture: iatrogenic causes and non-iatrogenic causes [17]. Iatrogenic causes include structural tooth loss, effect of chemicals and intra-canal medicament, and effects of restorations. The non-iatrogenic causes include primary causes, such as a history of recurrent pathology and anatomical position of the tooth, and secondary causes, such as the effect of dentinal tissue aging. Ultimately, these causes affect the ability of the teeth to withstand stress concentrations during loading. Practitioners should carefully scrutinize restorative material decisions and the extent of tooth structure removed when assigning prognosis to a previously root-canal-treated tooth. By considering these factors, one may be able to more accurately predict and assess the risk of tooth fracture. 

Although a high success rate (86–98%) of root canal treatment has been reported, several causes, including inappropriate mechanical debridement, persistence of bacteria in the canals and apex, poor obturation quality, over and under extension of the root canal filling, and coronal leakage, may still result in a failed outcome [18]. The majority of the teeth included in the current study (80%) were posterior teeth, which are normally more difficult to treat in terms of endodontic access, complete debridement and disinfection of all canals, and proper obturation than anterior teeth. These factors may have been more pronounced in certain provider demographics (i.e., general practitioners vs. endodontists), but the nature of the current study did not allow for this type of data analysis. Due to these challenges, the flare-ups of endodontic lesions or incomplete root canal treatment are more likely to occur, potentially resulting in the tooth extraction of teeth with even a periodontally favorable prognosis. 

Root-canal-treated teeth are often restored with direct or indirect restorations, which can often mask recurrent caries until a tooth becomes non-restorable and must be extracted. This result is consistent with a study by Frisk et al., which analyzed a total of 9779 teeth and found a significant association between root-canal-treated teeth and recurrent caries [19]. It has been reported that the *S. mutans* count and de novo plaque formation were significantly higher in root-canal-treated teeth than in vital teeth, which resulted in a higher risk of recurrent caries [20]. Therefore, clinicians should thoroughly evaluate the marginal fit of existing restorations for root-canal-treated teeth with even a favorable periodontal prognosis.

While the use of anti-depressants, i.e., selective serotonin reuptake inhibitors (SSRIs), has been strongly implicated in implant complications, limited evidence exists that link it to periodontal degradation in natural dentition [21,22]. Our finding suggests that patients using anti-depressants were over four times more likely to lose favorable teeth in the short term. A recent study also reported that increased periodontal inflammatory parameters, increased pocket depths, and clinical attachment loss were more significant in patients that used anti-depressants than the controlled subjects [23]. Although the mechanism of this outcome is still not fully understood, patients with mental health issues are often at a greater risk of oral health problems due to poor nutrition, inadequate oral hygiene, and poor compliance [24]. 

In terms of the initial periodontal status, our data showed that patients with an initial periodontal diagnosis of Stage III/IV periodontitis [25] were nearly three times more likely to lose their favorable teeth in the short term than patients with Stage I/II periodontitis. Stage III/IV periodontitis is defined as severe periodontal disease with a loss of attachment of 5 mm or more; while on an individual tooth basis, a favorable tooth may exist under these conditions, our data suggest that severe periodontal involvement in certain areas of the mouth has a detrimental impact on other teeth with even a favorable prognosis. This outcome is consistent with a recent study which concluded that higher concomitant staging and grading corresponded with a greater risk for tooth loss due to periodontal disease [26]. In addition, our study found that teeth with an initial pocket depth greater than or equal to 5 mm were over four times more likely to be extracted than teeth that had pocket depths less than 4 mm. This result validates long-term clinical data, indicating that teeth with deep probing depths are at significantly higher odds of being lost [27,28]. 

Frequent supportive periodontal therapy is often considered as one of the most pivotal factors for preventing tooth loss and can help minimize disease progression for patients with poor plaque control [29,30]. Our study found that the teeth that were infrequently maintained were over three times more likely to be lost than those that were well maintained. This is consistent with a series of studies conducted by Becker et al. [4,8,9]. The authors found an annual tooth loss of 0.11, 0.22, and 0.36 for the treated with maintenance, treated without maintenance, and untreated groups, respectively. Our study finding reinforces the importance of frequent dental cleanings (at least two times per year) to minimize the possibility of tooth loss due to periodontal disease.

Furcation involvement has been a challenge for maintaining periodontally compromised teeth. Even in a well-maintained patient population, teeth with furcation involvement have been shown to be significantly more likely to be lost [31]. In addition, even with surgical access, complete debridement of the furcation area of a tooth is not predictable [32]. Our study showed that the periodontal condition of a seemingly favorable tooth with incipient furcation involvement can still rapidly deteriorate. Therefore, the influence of incipient furcation involvement on the future periodontal prognosis may be underestimated. Clinicians should take into account the existing furcation involvement more seriously while planning future restorative treatment.

In terms of the percentage of teeth lost in relation to time (Figure 1), our study found that less than 20% of tooth loss occurred within the first two years and a large number of teeth were lost among all categories after 44 months of follow-up. The slightly higher tooth loss in the first two years due to periodontal reasons seems contrary to what one would expect to find in a study of this nature. Using a prognostication system centered around periodontal factors, it would be expected that teeth deemed favorable would be lost to non-periodontal reasons and may not have been initially identified. Nonetheless, this highlights the occasional unpredictability of disease progression of periodontitis. Jeffcoat and Reddy proposed three models of periodontal disease progression: a linear model, a burst model, and a model that featured sites with spontaneous exacerbations and remissions [33]. Though both the burst model and the exacerbation and remission rarely occur, both are defined by their seemingly spontaneous and significant uptick in attachment loss, which may represent the early loss of these periodontally favorable teeth shown in Figure 1. Another intriguing finding was the spontaneous exacerbation of tooth loss for all categories after 44 months of follow-up. This timeline may represent a key turning point where teeth begin to shift from a favorable prognosis to other poorer prognosis categories. As described in McGuire’s prognostication system [6], 15% of teeth with an initial good prognosis shifted to a worse prognosis after 5 to 8 years. Therefore, it is reasonable to anticipate that a very small percentage (2.1% reported in the current study) of the initially favorable teeth deteriorated to a hopeless prognosis, starting at the 44-month mark. More longitudinal data are needed to further investigate the percentage of tooth loss in relation to time.

There are several limitations for the current study. First, due to the retrospective nature of this study, some clinical parameters, i.e., oral hygiene status and occlusion, could not be assessed. Second, due to the structure of care within a dental school setting, there might be variability in patient care due to provider experiences. Third, for a study of this scope, a larger number of both patients and teeth would need to be provided for a more robust data analysis, especially when considering the categories of endodontic involvement and tooth fracture. 

## 5. Conclusions

Within the limitations of this study, it can be concluded that previously root-canal-treated teeth present a significantly higher OR for early loss of favorable teeth due to caries, endodontic reasons, or fracture. In addition, patients with anti-depressant use, sporadic maintenance, initial probing depths ≥5 mm, and furcation involvement demonstrate significantly higher OR of tooth loss due to periodontal disease, even for initially seemingly favorable teeth.

## Figures and Tables

**Table 1:** Demographic data of the participants.

Gender	Male: 26 (52%)	Female: 24 (48%)
Age	57.46 ± 13.74 years old, ranging 30 to 81	
Smoking Status	Yes: 7 (14.0%)	No: 43 (86.0%)
Diabetes	Yes: 8 (16.0%)	No: 42 (84.0%)
Osteoporosis	Yes: 3 (6%)	No: 47 (94%)
Anti-depressants	Yes: 4 (8%)	No: 46 (92%)
Initial Periodontal Diagnosis	Stage I	4 (8%)
	Stage II	10 (20%)
	Stage III	32 (64%)
	Stage IV	4 (8%)

**Table 2:** Features of the extracted teeth for each category.

Reason of Loss	Caries N = 49	2: Periodontal Disease N = 35	3: Endodontic Reasons N = 11	4: Fracture N = 16
Tooth location *	Max ant: 5 Max post: 21 Mand ant: 3 Mand post: 20	Max ant: 3 Max post: 19 Mand ant: 4 Mand post: 9	Max ant: 3 Max post: 4 Mand ant: 1 Mand post: 3	Max ant: 3 Max post: 8 Mand ant: 0 Mand post: 5
Initial crown-to-root ratio	0.47	0.48	0.53	0.45
Initial periodontal diagnosis	Stage I/II: 14 Stage III/IV: 35	Stage I/II: 0 Stage III/IV: 35	Stage I/II: 5 Stage III/IV: 6	Stage I/II: 5 Stage III/IV: 11
Initial pocket depth (mm)	Mean: 3.94 SD: 1.18	Mean: 5.57 SD: 1.65	Mean: 3.91 SD: 1.14	Mean: 3.81 SD: 1.22
Number of cleanings per year	Mean: 1.28 SD: 0.77	Mean: 1.16 SD: 0.63	Mean: 1.55 SD: 0.69	Mean: 1.3 SD: 0.70
Existing restorations	None: 10 Direct: 24 Indirect: 15	None: 16 Direct: 13 Indirect: 6	None: 1 Direct: 6 Indirect: 4	None: 8 Direct: 2 Indirect: 6
Root-canal-treated teeth	Yes: 8 No: 41	Yes: 4 No: 31	Yes: 2 No: 9	Yes: 9 No: 7
Furcation involvement	Yes: 4 No: 23	Yes: 10 No: 5	Yes: 0 No: 5	Yes: 2 No: 2

* Max: maxillary, Mand: mandibular, ant: anterior, post: posterior.

**Table 3:** Risk indicators of tooth loss for each category using a linear mixed model.

	Caries	Periodontal Disease	Endodontic Reason	Fracture	*p* Value
Smoking	NS	NS	NS	NS	0.1687
Diabetes	NS	NS	NS	NS	0.2144
Osteoporosis	NS	NS	NS	NS	0.2769
Anti-depressants	NS	OR 4.28 4.14–4.42	NS	NS	<0.0001
Crown-to-root ratio	NS	NS	NS	NS	0.2574
Initial periodontal diagnosis (Stage I or II vs. Stage III or IV)	NS	OR 2.66 2.43–2.89	NS	NS	<0.0001
Initial pocket depth (<4 mm vs. ≥5 mm)	NS	OR 4.32 3.83–5.12	NS	NS	<0.0001
Number of cleanings per year (<2 times vs. ≥2 times)	NS	OR 3.13 1.18–5.08	NS	NS	0.0022
Existing restorations	NS	NS	NS	NS	0.3533
Root-canal-treated teeth	OR 3.61 3.39–4.23	NS	OR 3.86 3.63–4.09	OR 2.53 2.24–2.83	<0.0001
Furcation involvement (for molars only)	NS	OR 1.93 1.42–2.44	NS	NS	0.0006

NS: not statistically significant.

## Data Availability

Data are available on request due to restrictions.
